# Supplementary material for: A large-scale field study examining effects of exposure to clothianidin seed-treated canola on honey bee colony health, development, and overwintering success
Source: PeerJ. 2014 Oct 30;2:e652. doi: 10.7717/peerj.652 (PMC4217196; doi:10.7717/peerj.652)
Supplement: Table S1 — Percentage of canola (Brassica napus) (±SD), corn (Zea mays), soybean (Glycine max), and other pollen collected by honey bees during exposure to 2-ha canola fields for two weeks. [file peerj-02-652-s001.docx]

| **Treatment** | **Site** | **Pollen type** | **Week 1** | **Week 2** |
| --- | --- | --- | --- | --- |
| Control | 1 | Canola | 95.0 | 2.0 |
|  |  | Corn | 0.0 | 0.0 |
|  |  | Soybean | 0.0 | 0.0 |
|  |  | Other | 5.0 | 98.0 |
| Control | 2 | Canola | 85.9 | 1.0 |
|  |  | Corn | 0.0 | 5.0 |
|  |  | Soybean | 0.0 | 0.0 |
|  |  | Other | 14.1 | 94.0 |
| Control | 3 | Canola | 59.3 | 15.7 |
|  |  | Corn | 0.0 | 0.0 |
|  |  | Soybean | 0.0 | 0.0 |
|  |  | Other | 40.7 | 84.3 |
| Control | 6 | Canola | 97.8 | 86.0 |
|  |  | Corn | 0.0 | 0.0 |
|  |  | Soybean | 0.0 | 0.0 |
|  |  | Other | 2.2 | 14.0 |
| Control | 10 | Canola | 86.5 | 83.0 |
|  |  | Corn | 0.0 | 0.7 |
|  |  | Soybean | 0.0 | 0.0 |
|  |  | Other | 3.5 | 16.3 |
| Treated | 4 | Canola | 85.5 | 3.7 |
|  |  | Corn | 0.0 | 0.3 |
|  |  | Soybean | 0.0 | 0.0 |
|  |  | Other | 14.5 | 96.0 |
| Treated | 5 | Canola | 89.4 | 6.3 |
|  |  | Corn | 0.0 | 6.0 |
|  |  | Soybean | 0.0 | 0.0 |
|  |  | Other | 10.6 | 87.7 |
| Treated | 7 | Canola | 99.8 | 94.0 |
|  |  | Corn | 0.0 | 0.0 |
|  |  | Soybean | 0.0 | 0.0 |
|  |  | Other | 0.2 | 6.0 |
| Treated | 8 | Canola | 94.7 | 77.3 |
|  |  | Corn | 0.0 | 0.3 |
|  |  | Soybean | 0.0 | 0.0 |
|  |  | Other | 5.3 | 22.4 |
| Treated | 9 | Canola | 85.7 | 92.7 |
|  |  | Corn | 0.0 | 0.7 |
|  |  | Soybean | 0.0 | 0.0 |
|  |  | Other | 14.3 | 6.6 |
