# Supplementary material for: A large-scale field study examining effects of exposure to clothianidin seed-treated canola on honey bee colony health, development, and overwintering success
Source: PeerJ. 2014 Oct 30;2:e652. doi: 10.7717/peerj.652 (PMC4217196; doi:10.7717/peerj.652)
Supplement: Table S2 — Wild pollen types recovered from honey bee colonies outfitted with pollen traps during and after placement in canola fields in southern Ontario. [file peerj-02-652-s002.docx]

| **Name of pollen type** | **Family** | **Common names** | **Representative species or genera** |
| --- | --- | --- | --- |
| *Centaurea* type *nigra* | Asteraceae | Composites, knapweed | *Centaurea nigra, C. nemoralis* |
| *Cornus* type *stolonifera* | Cornaceae | Dogwood family | *Cornus stolonifera, C. alternifolia, C. rugosa* |
| Type *Allium* | Liliaceae | Lily family, wild onion etc. | *Allium, Polygonatum, Ruscus* |
| Type *Aster* / *Solidago* | Asteraceae | Asters and other wildflowers | *Aster, Solidago, Helianthus, Rudbeckia, Tussilago, Petasites, Arnica, Filago* |
| Type *Chrysanthemum* | Asteraceae | Chrysanthemum, wild flowers | *Chrysanthemum, Achillea, Tanacetum, Anthemis, Cotula, Matricaria, Onopordum, Pulicaria* |
| Type *Daucus* | Apicaceae | Wild carrot, parsley family | *Daucus, Pastinaca, Angelica, Aethusa,* and possibly *Carum* |
| Type *Melilotus* | Fabaceae | Legumes (sweet clover, herbs, loco weeds) | *Melilotus, Astragalus, Ononis, Oxytropis* |
| *Type Mentha* | Lamiaceae | Mints | *Mentha, Lycopus, Thymus, Origanum, Salvia, Monarda* |
| Type *Prunella* | Lamiaceae | Herbs, self heal | *Prunella, Glechoma, Nepeta, Agastache* |
| Type *Taraxacum* | Asteraceae | Dandelions, etc. | Liguliflorae: *Taraxacum, Cichorium, Hieracium, Lactuca, Sonchus, Tragopogon, Arnoseris, Crepis, Hypochoeris, Lapsana, Leontodon, Picris* |
| Type *Trifolium* H / R | Fabaceae | White clovers | *Trifolium hybridum, T. repens, T. agrarium, T. arvense; Medicago lupulina* |
| Type *Trifolium pratense* | Fabaceae | Red clover | *Trifolium pretense* |
| Type *Typha angustifolia* | Typhaceae | Narrow-leaf cattail | *Typha angustifolia, Sparganium* |
